# The association between political orientation and political knowledge in 45 nations

**DOI:** 10.1038/s41598-024-53114-z

**Published:** 2024-01-31

**Authors:** Jonas De keersmaecker, Katharina Schmid, Chris G. Sibley, Danny Osborne

**Affiliations:** 1 Ghent University, Ghent, Belgium; 2 Esade Business School, Universitat Ramon Llull, Barcelona, Spain; 3 University of Auckland, Auckland, New Zealand

**Keywords:** Psychology, Human behaviour

## Abstract

Political knowledge is crucial for well-functioning democracies, with most scholars assuming that people at the political extremes are more knowledgeable than those at the center. Here, we adopt a data-driven approach to examine the relationship between political orientation and political knowledge by testing a series of polynomial curves in 45 countries (*N* = 63,544), spread over 6 continents. Contrary to the dominant perspective, we found no evidence that people at the political extremes are the most knowledgeable about politics. Rather, the most common pattern was a fourth-degree polynomial association in which those who are moderately left-wing and right-wing are more knowledgeable than people at the extremes and center of the political spectrum. This pattern was especially, though not exclusively, prevalent in Western countries. We conclude that the relationship between political orientation and political knowledge is more context-dependent and complex than assumed, and caution against (implicit) universal conclusions in social sciences.

## Introduction

Understanding citizens’ factual knowledge about politics has long been of interest to social and behavioral scientists, as well as politicians and policy makers—and for good reason. Political knowledge critically shapes people’s political attitudes and behavior^1,2^ and acceptance of democratic principles^3^, while also allowing citizens to identify the policy positions that best resonate with their underlying traits^4,5^ and values^6^. Yet much of this work reveals that the average citizen lacks a basic understanding of politics^7–9^. This has led scholars to investigate potential ideological asymmetries in political knowledge^10^. Central to this literature is a foundational question that also has critical implications for policy making: Are uninformed citizens particularly susceptible to extreme left-wing or right-wing political orientations? Or do those more knowledgeable about politics endorse more extreme left-wing or right-wing ideologies? To date, the relationship between political orientation and factual political knowledge is poorly understood and little is known about the variability of this relationship across nations.

Current literature assumes that people at the political extremes are more knowledgeable about politics than moderates^1,11^, with studies demonstrating a positive association between political extremism and political knowledge/sophistication^10–14^. However, the evidence rests on a limited number of studies, most of which originate from the United States. Moreover, past studies mostly employed a limited set of statistical models to study the relationship between political orientation and knowledge.

The present paper makes two key advances that address these lacunae. First, we examine the relationship between political orientation and political knowledge using unprecedented cross- cultural data from 45 countries, spread over six continents. Second, we use robust statistical analyses to overcome limitations of earlier studies.

Investigations in behavioral sciences must consider the socio-cultural context, rather than relying solely on Western samples that limit generalizability^15^, particularly for political issues. Indeed, the context theory of the ideology-personality interface^10^ argues that political extremity is defined within a specific cultural-historical context and is therefore only meaningful in relative, rather than absolute, terms. Moreover, the relationship between political attitudes and cognitive characteristics depends on the psychological functions political attitudes serve, which likely vary by the culture in which a person is embedded.

Consistent with context theory^10^, ample evidence demonstrates that the antecedents and consequences of political ideology are context- and culture-specific. For example, the relationships political orientation has with personality^16^, epistemic motivations^17^, wellbeing^18^, and outgroup attitudes^19^ all vary by the socio-cultural context. It is therefore imperative to examine the relationship between political orientation and political knowledge across nations.

Investigations into the relationship between political orientation and political knowledge also must utilize robust statistical models to allow for diverse patterns to emerge. Past research mainly tested only two possibilities: whether the relationship between political orientation and political knowledge is (a) linear or (b) quadratic (i.e., a U-shaped curve)^20^. Moreover, political extremity is often operationalized simply as a difference score from the political center^13^. Although these statistical approaches can be informative, they risk oversimplifying complex associations. For example, a quadratic association might fit the data better than a simple linear association, but this does not imply that a quadratic function is the *best* fitting model.

Recently, scholars examining political behavior and attitudes have advocated for more research utilizing a data-driven, bottom-up approach since testing a single model increases imprecision^21^. The present research adopts such a data-driven approach by testing a consecutive series of polynomial regressions modeling the relationship between political orientation and factual political knowledge in 45 nations, thereby allowing complex relationships to emerge. Because we provide the first ever examination of this research question across such diverse socio-political contexts, we do not offer predictions for which, if any, specific polynomial will best represent the association between political knowledge and political orientation. Likewise, we abstain from conjecturing how this association will differ across nations. Rather, we provide core empirical foundations for future research and policymaking by identifying the nature of this relationship across a range of diverse national contexts.

## Method

We draw upon public opinion data using nationally representative samples from 45 countries. Two of these datasets (namely, data from Spain and New Zealand) were designed and collected by our research team, and we supplemented these data with 43 additional publicly available datasets. For the latter samples, we conducted an online search for nationally representative datasets from different countries that contained both a measure of political orientation and a factual political knowledge test. For each country, we selected no more than one sample. If a given data source contained multiple datasets (e.g., the American National Election Survey), we selected the most recently available dataset. Whenever several datasets were available in the same country (e.g., American National Election Survey and Comparative Study of Electoral Systems), we selected the dataset with the most reliable political knowledge measure. See https://osf.io/gbp6z/ for the specific source of each dataset, year of data collection, and interview mode.

In all samples, political orientation was measured with a single-item Likert scale ranging from left (0 or 1, depending on the country) to right (7 or 10, depending on the country). Political knowledge was measured with a multi-item test (ranging from 3 to 21 items) of general political facts. An example item reads: ‘Who is the current minister of finance in [country]?’ OSF contains the specific items of the political knowledge test for each country, as well as the statistical properties of the political orientation and political knowledge measures. In total, we identified datasets from 45 countries, resulting in a combined sample of 63,544 participants.

### Ethics statement

The research was conducted according to the ethical rules presented in the General Ethical Protocol of the Faculty of Psychology and Educational Sciences of Ghent University.

### Statement of informed consent

The data analyzed in the manuscript are publicly available opinion survey data. The datasets of Spain and New Zealand were designed and collected by the authors of the manuscript. Participants provided their informed consent prior to their participation in the study.

## Results

To examine the association between political ideology and political knowledge, we used the *poly* function from the *stats* package in *R*^22^ to sequentially run a series of five orthogonal polynomial regressions in each country. We analyzed the data in every country separately rather than analyzing a multi-level model since the latter approach overlooks country-specific nuances. Specifically, the proportion of correct answers on the political knowledge test (y) was regressed onto the z-score of political ideology (x) from the 1st degree up to the 5th degree orthogonal polynomial:

- Model 1: y = b0 + b_1_x
- Model 2: y = b0 + b_1_x + b_2_x^2^
- Model 3: y = b0 + b_1_x + b_2_x^2^ + b_3_x^3^
- Model 4: y = b0 + b_1_x + b_2_x^2^ + b_3_x^3^ + b_4_x^4^
- Model 5: y = b0 + b_1_x + b_2_x^2^ + b_3_x^3^ + b_4_x^4^ + b_5_x^5^

Orthogonal coding of the polynomials ensures that a higher order term only captures variance that is not explained by a lower order term. For each country, the model that significantly (*p* < 0.050) explained the most variance was selected. In cases where two or more models did not differ significantly in their explained variance, the most parsimonious model was selected. When available in the given dataset, sample weights were used.

**Figure 1:**
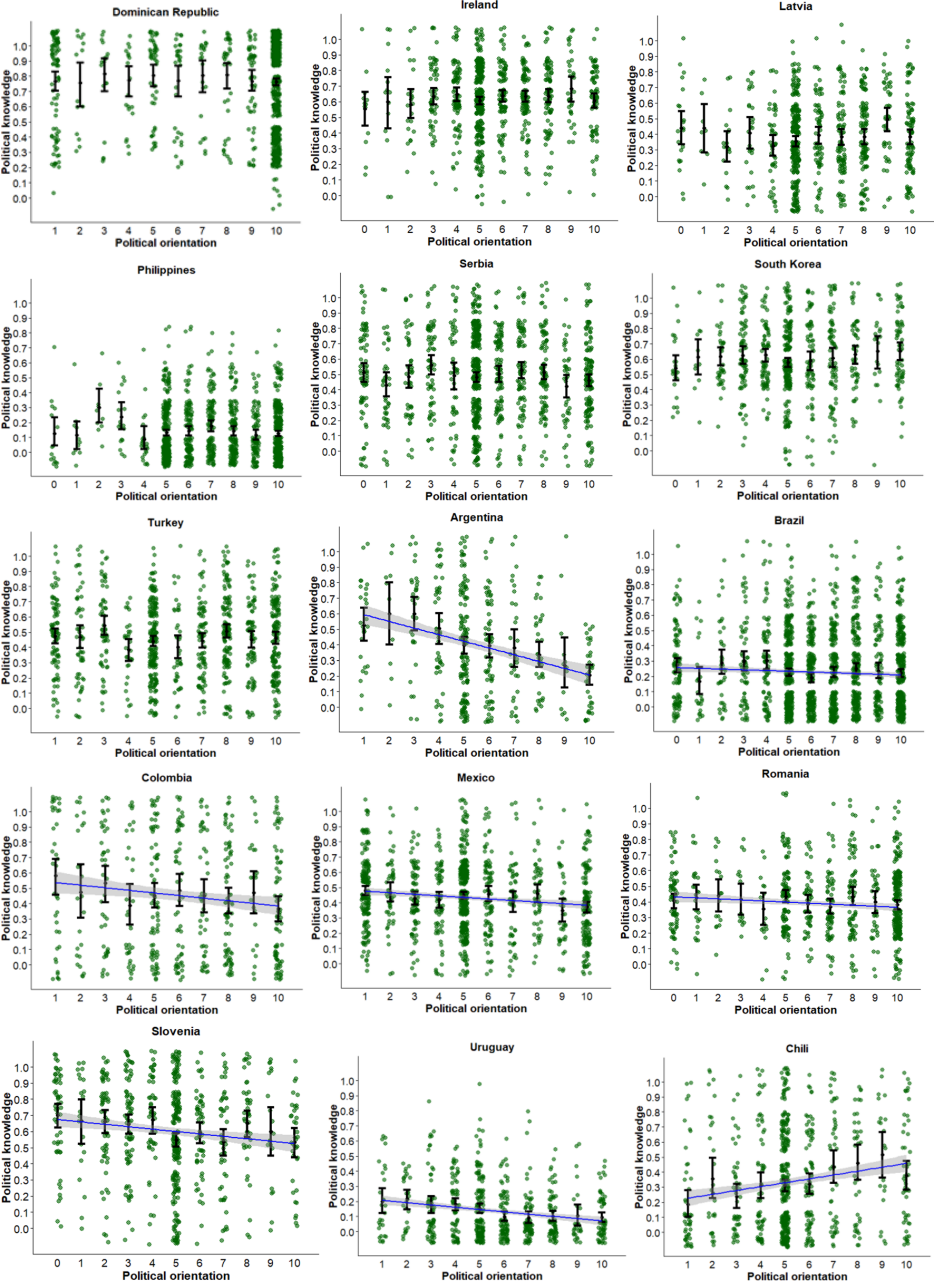

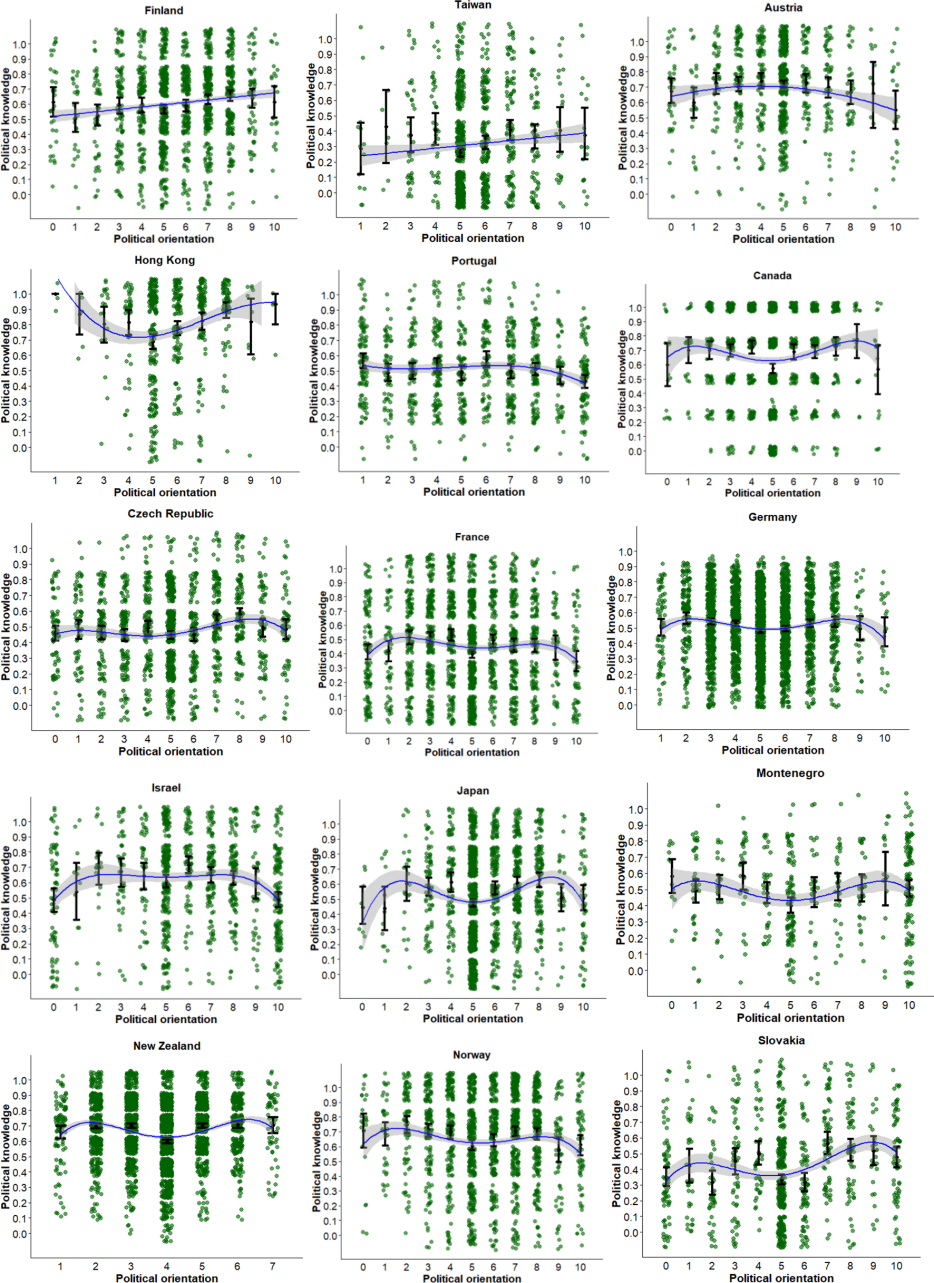

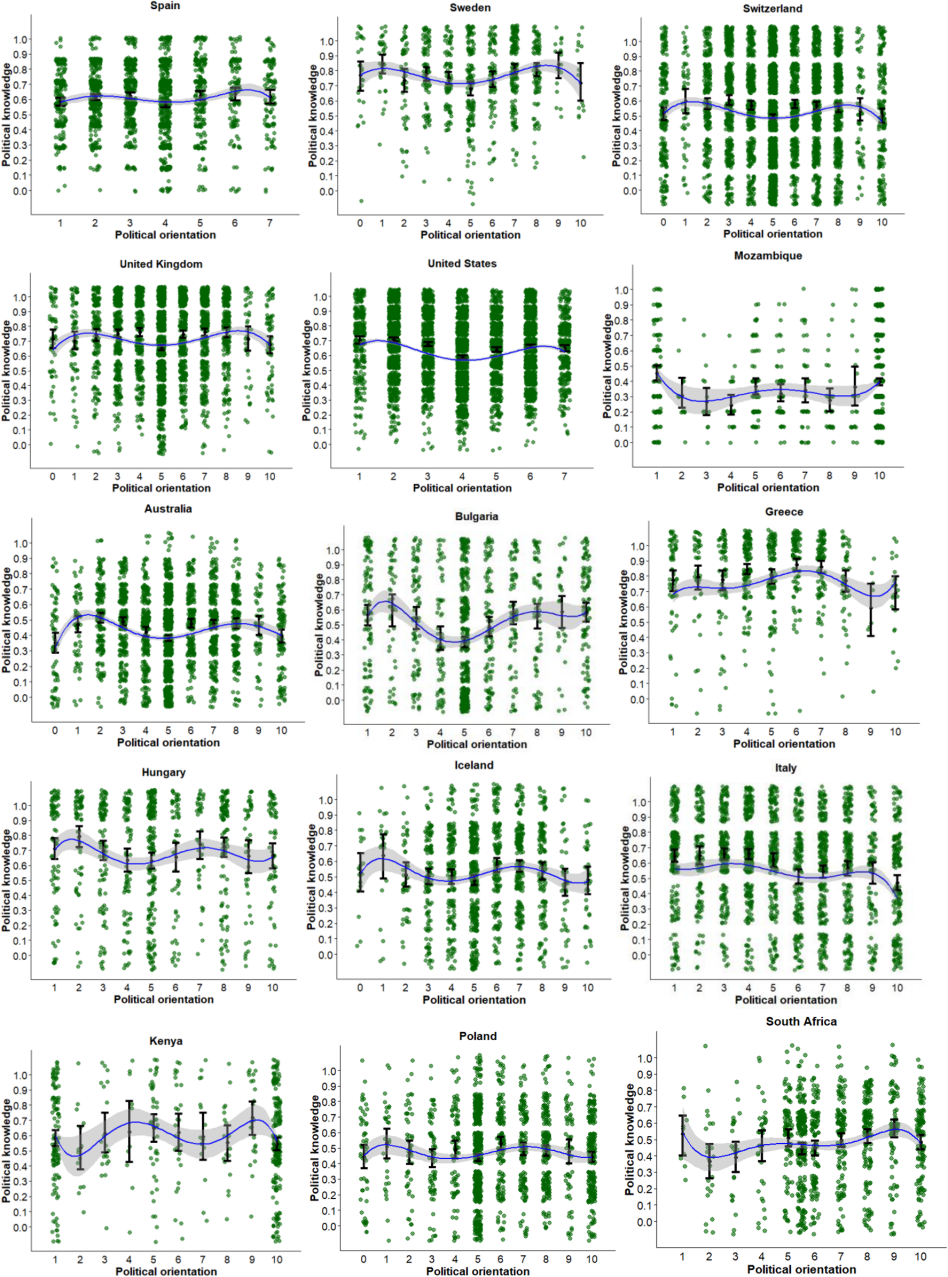
Visualization of the relationship between political orientation and political knowledge. Note. The blue curve is the best fitting (polynomial) regression line, the gray shade represents its 95% confidence interval. For each value on the political orientation dimension, the dot with error bars represents the mean and its 95% confidence interval.

Table 1 presents the estimated parameters, standard errors, and adjusted *R*^2^ of the best fitting model for each country (also see Fig. 1). As shown here, there is sizeable heterogeneity in the association between political orientation and political knowledge across countries. Therefore, we ordered countries in the table and figure from the absence of a significant association between political orientation and knowledge up to countries where a 5^th^ degree orthogonal polynomial best-explained these data.

**Table 1:** The association between political orientation and political knowledge in 45 countries.

Country (sample size)	b1		b2		b3		b4		b5		*Adj. R*^2^
	*b*	*p*	*b*	*p*	*b*	*p*	*b*	*p*	*b*	*p*	
	*se*		*se*		*se*		*se*		*se*		
Dominican Rep. (1029)	− 0.005	0.571									0.000
	0.009										
Ireland (904)	− 0.004	0.577									0.000
	0.007										
Latvia (787)	0.010	0.266									0.000
	0.009										
Philippines (1179)	− 0.008	0.141									0.001
	0.005										
Serbia (1094)	− 0.007	0.400									0.000
	0.008										
South Korea (717)	0.013	0.149									0.002
	0.009										
Turkey (974)	− 0.001	0.877									0.000
	0.008										
Argentina (443)	− 0.096	< 0.001									0.090
	0.014										
Brazil (1765)	− 0.013	0.022									0.002
	0.006										
Chile (769)	0.057	< 0.001									0.026
	0.012										
Colombia (537)	− 0.047	0.007									0.012
	0.017										
Finland (1388)	0.033	< 0.001									0.014
	0.007										
Mexico (1119)	− 0.028	< 0.001									0.014
	0.007										
Romania (714)	− 0.023	0.007									0.009
	0.008										
Slovenia (667)	− 0.038	< 0.001									0.015
	0.012										
Taiwan (1000)	0.029	0.010									0.006
	0.011										
Uruguay (651)	− 0.036	< 0.001									0.036
	0.007										
Austria (881)	− 0.366	0.174	− 0.830	0.002							0.010
	0.269		0.268								
Hong Kong (624)	1.072	< 0.001	1.090	< 0.001	− 0.654	0.036					0.040
	0.322		0.313		0.312						
Portugal (919)	− 0.635	0.011	− 0.580	0.018	− 0.555	0.023					0.016
	0.248		0.245		0.243						
Canada (1243)	0.288	0.349	0.984	0.002	0.031	0.921	− 0.843	0.006			0.012
	0.307		0.313		0.311		0.308				
Czech Republic (1426)	0.804	0.001	0.434	0.079	− 0.503	0.044	− 0.740	0.003			0.016
	0.247		0.247		0.249		0.248				
France (1916)	− 0.446	0.155	− 0.693	0.028	0.381	0.224	− 1.214	< 0.001			0.010
	0.313		0.314		0.313		0.311				
Germany (3295)	− 0.325	0.154	0.542	0.019	0.044	0.848	− 1.241	< 0.001			0.010
	0.228		0.232		0.231		0.231				
Israel (924)	− 0.278	0.344	− 1.857	< 0.001	− 0.311	0.291	− 0.736	0.012			0.046
	0.294		0.294		0.294		0.294				
Japan (1563)	0.792	0.011	0.381	0.233	− 0.749	0.019	− 1.776	< 0.001			0.028
	0.312		0.319		0.319		0.311				
Montenegro (457)	− 0.007	0.979	0.613	0.015	− 0.225	0.368	− 0.583	0.022			.017
	0.249		0.251		0.250		0.253				
Mozambique (890)	− 0.094	0.718	1.158	< 0.001	− 0.191	0.459	0.648	0.012			0.025
	0.259		0.259		0.259		0.259				
New Zealand (3428)	0.019	0.926	1.408	< 0.001	0.401	0.045	− 1.650	< 0.001			0.033
	0.200		0.200		0.200		0.200				
Norway (1663)	− 0.647	0.033	0.061	0.838	− 0.272	0.363	− 1.106	< 0.001			0.009
	0.304		0.298		0.299		0.300				
Slovakia (879)	1.418	< 0.001	0.786	0.009	− 0.074	0.802	− 1.207	< 0.001			0.050
	0.301		0.298		0.295		0.296				
Spain (1957)	0.185	0.396	0.236	0.279	0.306	0.161	− 0.747	< 0.001			0.006
	0.218		0.218		0.218		0.218				
Sweden (792)	0.263	0.266	0.632	0.008	− 0.339	0.152	− 0.840	< 0.001			0.024
	0.236		0.236		0.236		0.236				
Switzerland (4278)	− 0.314	0.351	0.576	0.086	− 0.376	0.256	− 2.151	< 0.001			0.011
	0.337		0.335		0.330		0.325				
United Kingdom (3229)	0.269	0.323	0.642	0.018	− 0.037	0.892	− 1.655	< 0.001			0.013
	0.272		0.272		0.270		0.269				
United States (6004)	− 0.870	< 0.001	2.428	< 0.001	− 0.036	0.870	− 2.132	< 0.001			0.037
	0.221		0.217		0.220		0.219				
Australia (2375)	− 0.049	0.842	0.505	0.039	0.160	0.516	− 2.122	< 0.001	0.629	0.009	0.038
	0.245		0.245		0.246		0.240		0.240		
Bulgaria (1058)	0.461	0.189	2.071	< 0.001	− 0.766	0.029	− 1.246	< 0.001	0.968	0.006	0.051
	0.351		0.351		0.351		0.351		0.351		
Greece (672)	0.787	< 0.001	− 0.625	0.004	− 0.290	0.189	0.335	0.149	0.567	0.012	0.033
	0.224		0.214		0.221		0.232		0.225		
Hungary (878)	− 0.321	0.399	0.608	0.110	− 0.453	0.232	− 0.579	0.118	0.839	0.022	0.009
	0.380		0.380		0.378		0.370		0.365		
Iceland (1266)	0.208	0.470	0.021	0.941	− 0.885	0.002	− 0.334	0.246	0.793	0.006	0.011
	0.288		0.288		0.288		0.288		0.288		
Italy (2086)	− 2.217	< 0.001	− 1.186	< 0.001	− 0.267	0.437	− 1.039	0.003	− 0.874	0.011	0.035
	0.336		0.342		0.343		0.344		0.342		
Kenya (503)	− 0.201	0.503	− 0.405	0.181	− 0.187	0.546	− 0.002	0.995	− 0.854	0.005	0.011
	0.300		0.302		0.309		0.309		0.303		
Poland (1634)	0.042	0.877	− 0.181	0.499	− 0.668	0.012	− 0.434	0.102	0.568	0.032	0.006
	0.268		0.268		0.265		0.266		0.265		
South Africa (967)	0.599	0.020	− 0.103	0.692	− 0.483	0.058	− 0.192	0.448	− 0.698	0.007	0.012
	0.258		0.260		0.254		0.252		0.258

### Absence of an association

In several countries, political orientation did not significantly predict political knowledge. The absence of a significant association between political orientation and political knowledge emerged in geographical regions around the world (7 out of 45 countries: Dominican Republic, Ireland, Latvia, Philippines, Serbia, South Korea, and Turkey).

### Linear relationship

An equally common pattern was a linear relationship between political orientation and political knowledge (10 out for 45 countries). We found both positive (Chile, Finland, and Taiwan) and negative (Argentina, Brazil, Colombia, Mexico, Romania, Slovenia, and Uruguay) linear relationships. The linear pattern was especially prevalent in Latin American countries, but also emerged in some European and Asian countries.

### Quadratic relationship

Austria was the only country in which a quadratic relationship emerged between political orientation and knowledge; people at the political center were relatively more knowledgeable than people who placed themselves at the extremes.

### Cubic relationship

We found a cubic relationship between political orientation and knowledge in two countries. The pattern reflects a non-symmetric U-shape in Hong Kong in which those at the extremes are more knowledgeable than those at the center, and extreme left-wing individuals tend to be slightly more knowledgeable than extreme right-wing individuals. But in Portugal, extreme left-wing individuals and centrists tend to have more political knowledge than extreme right-wing individuals (although the differences are small).

### Quartic relationship

The most frequently observed pattern (15 out of 45 countries: Canada, Czech Republic, France, Germany, Israel, Japan, Montenegro, New Zealand, Norway, Slovakia, Spain, Sweden, Switzerland, United Kingdom and United States) was a fourth-degree polynomial curve reflecting an inverted W-shape. Specifically, individuals who considered themselves as moderately left-wing or moderately right-wing were more politically knowledgeable than individuals who placed themselves in the center of the political spectrum, as well as those at the extremes. The majority of, albeit not all, countries that showed this pattern are Western countries. The reversed pattern was found in Mozambique, as those on the centre, the extreme left and extreme right were most knowledgeable about politics.

### Quintic relationship

Finally, in several countries (9 out of 45 countries: Australia, Bulgaria, Greece, Hungary, Iceland, Italy, Kenya, Poland, and South Africa), we found a fifth-degree polynomial cruve between political orientation and political knowledge. These countries reflect considerable heterogeneity, and the association between political orientation and political knowledge is asymmetric among the political center. Like the other patterns, the quintic association emerged across geographical regions.

Considering that age, gender, and education correlate with both political orientation^23^ and political knowledge^1^, we also examined the relationship between political orientation and political knowledge after adjusting for these covariates. For Kenya, Poland, Hungary, Brazil, and Taiwan, (the polynomial terms of) political orientation did not significantly predict political knowledge after adding these covariates to the model. For Bulgaria (quartic), Hong Kong (quadratic), Israel (quadratic), Canada (quadratic), and Portugal (linear), a model with fewer polynomial terms provided a better fit to these data. Finally, a significant quadratic pattern emerged for Ireland and Romania after adjusting for age, gender, and education. OSF contains the country-specific results of these additional analyses.

## Discussion

We provided a comprehensive and statistically robust examination of the relationship between political orientation and political knowledge across 45 nations. Contrary to prevalent assumptions^12^, our investigation did not reveal that people at the political extremes are the most knowledgeable about politics. In fact, not a single one of the 45 national samples analyzed here displayed this widely-assumed quadratic association. Rather, our analyses demonstrate that the relationship between political orientation and political knowledge is complex and nuanced, and, importantly, that it varies across countries. A polynomial association of the fourth degree was the most prevalent pattern, indicating that people who are moderately left-wing and moderately right-wing have relatively higher levels of political knowledge than people at both the extremes and center of the political spectrum. This pattern was particularly, though not exclusively, prevalent in Western countries. Conversely, a positive or negative simple linear relationship emerged in several Latin American countries. But once again, several countries deviated from these patterns. Furthermore, political orientation was not related (neither linearly, nor curvilinearly) to political knowledge in several countries around the globe. These results converge with previous work showing that the antecedents and consequences of political ideology vary across contexts^18^, and illustrate the perils of (implicit) universal conclusions.

Political orientation predicted political knowledge in 38 out of 45 countries. However, the explained variance was generally low, indicating that political orientation is not a main antecedent of factual political knowledge (or vice versa). Thus, although US citizens located at the political extremes tend to feel superior about their beliefs on political issues^24^, they are not substantially more or less knowledgeable than moderates about politics.

The present investigation extended the existing literature by examining a wide range of possible patterns between political orientation and knowledge. One reason why prior research may have narrowly focused on determining whether the association between political orientation and knowledge is merely linear or quadratic is that the prevalent discourse in the social sciences centers around questions of ideological symmetry or asymmetry, seeking to understand whether left-wing and right-wing individuals differ, for example, in partisan biases^25^, moral judgments^26^, and belief superiority^27^. Yet this lack of scholarly attention on people at the political center is unwarranted given that many citizens hold moderate views, and these citizens are especially important for candidate selection^11^. The present results corroborate the perils of focusing on the extremes and indicate that reducing the entire ideological spectrum into ‘the left’ and ‘the right’ is too simplistic and misses important nuances^21^.

Although the measures we used to operationalize political orientation and political knowledge were highly similar across contexts, these measures must be interpreted within their specific national context and historical zeitgeist^10^. For example, being a moderate today means something different than it did 50 years ago. Likewise, being a moderate in South Africa today represents distinct beliefs and opinions compared to contemporary moderates in the United States. Similarly, a specific factual political knowledge question (e.g., recalling the Minister of Finance) might be relatively easy or difficult to answer depending on the country-specific context and time. Accordingly, tests of measurement invariance for our multi-item measure of political knowledge are impractical, both because there is no reason to assume that knowledge would be similar in, for example, Taiwan and Uruguay, and because the items comprising our measure necessarily varied across datasets. Therefore, although our data are well equipped to compare the relationship between political knowledge and orientation within countries, these data do not allow for comparing average levels of these constructs between countries.

In this vein, a key avenue for future research will be to examine how changes within countries impact the relationship between political orientation and political knowledge. It will be valuable to understand, for example, whether changes in either the political orientation of the ruling government or the national economic situation impact the association between political orientation and knowledge. Studies that address these vital questions will increase understanding of how the broader socio-political context shapes the relationship between political orientation and political knowledge.

Although different domains of political knowledge are highly interrelated and can be captured by a single-factor model^1^, an interesting extension of the present investigation could examine the relationship between political orientation and domain-specific political knowledge. For example, political knowledge can vary by topic and temporal dimension^7^. Thus, the curvilinear associations identified here may only apply to general political knowledge measures. Future work could also incorporate longer measures of political knowledge than we had available for some countries. Short measures do, however, offer advantages in terms of costs and efficiency (key considerations in public opinions survey), and factual political knowledge can be measured with relatively few items^28^. Nevertheless, employing longer measures will help maximize validity and reliability^29^. Finally, some scholars argue that the political facts included in traditional political knowledge measures are peripheral to political decisions and insights. Consequently, they advocate for a mixed approach to measure political knowledge that incorporates surveys, interviews, and experiments^30^.

In the present investigation, political orientation was measured using a single item—a common practice in public opinion surveys^31^—because different domains of political orientation are typically intertwined and stem from overarching ideological beliefs^32^. Nevertheless, political orientation can also be conceptualized on two interrelated dimensions encompassing social-cultural and economic beliefs^33^. Investigating whether these dimensions exhibit varying relationships with (different aspects of) political knowledge will provide further insights. Additionally, considering the measurement of political orientation with a cluster of attitudes rather than the symbolic placement on a left–right scale may be valuable, as its utility for organizing political attitudes differs across contexts^34,35^.

Relatedly, it is important to note that extreme values on the left–right scale do not directly capture attitude intensity. For example, someone can support a left-wing policy like nationalizing the economy, albeit with low conviction. Therefore, in addition to measuring the direction of one’s political orientation, future research would benefit from also measuring the intensity of one’s attachment^36^. Indeed, research from the US reveals that attachment to, or identification with, one’s political orientation is a stronger predictor of cognitive inflexibility than the direction of one’s political orientation^37^. Political attachment also predicts the partisan endorsement of political misbeliefs^38^. Thus, measuring both the direction of, and attachment to, one’s political orientation may predict political knowledge in nuanced ways. For example, someone who places themselves on the left or right end of the political spectrum but is indifferent towards politics may have different levels of knowledge than someone who identifies as extreme left or extreme right and views politics as an important part of their self-concept^39^.

To conclude, political knowledge is one of the most valuable resources for citizens in a democracy^40^. The present investigation extended the theoretical debate of ideological symmetry and asymmetry among the political left and right by adopting a data-driven approach^21^ and showing that the relationship between political orientation and political knowledge is more context-dependent and nuanced than previously assumed. We hope to inspire scholars of different fields to use these results as a starting point for future research examining the factors that impact the relationship between political orientation and political knowledge both within and between nations.

## Data Availability

The data analyzed in the manuscript are publicly available opinion survey data, the source of the datasets can be found here: https://osf.io/gbp6z/
